# Lipopolysaccharide-induced murine lung injury results in long-term pulmonary changes and downregulation of angiogenic pathways

**DOI:** 10.1038/s41598-022-14618-8

**Published:** 2022-06-17

**Authors:** S. T. Tsikis, S. C. Fligor, T. I. Hirsch, A. Pan, L. J. Yu, H. Kishikawa, M. M. Joiner, P. D. Mitchell, M. Puder

**Affiliations:** 1 Vascular Biology Program, Boston Children’s Hospital, Harvard Medical School, Boston, MA 02115 USA; 2 Department of Surgery, Boston Children’s Hospital, Harvard Medical School, 300 Longwood Ave, Fegan 3, Boston, MA 02115 USA; 3 Institutional Centers for Clinical and Translational Research, Boston Children’s Hospital, Boston, MA 02115 USA

**Keywords:** Respiratory tract diseases, Respiration

## Abstract

Acute respiratory distress syndrome is the most severe form of acute lung injury (ALI) and is associated with significant mortality. Lipopolysaccharide (LPS)-induced injury is a valuable murine model of ALI but there is a paucity of data on lung regeneration and the role of angiogenic signaling involving vascular endothelial growth factor (VEGF). Eight-week-old male C57BL/6J mice were randomized to receive intratracheal instillation of either LPS or isovolumetric phosphate buffered saline as a vehicle control. Mice were observed at a single follow-up time-point that was either short-term (24 h or 4 days) or long-term (7 days or 4 weeks). On pulmonary function testing, LPS-treated mice had increased compliance at 4 weeks post-instillation, which correlated with decreased vascularization and with time-dependent, progressive decrease in alveolarization. Treadmill exercise tolerance testing demonstrated impaired performance at 24 h, 4 days and 4 weeks following LPS exposure. On lung protein analysis, LPS instillation decreased VEGF expression at up to 4 weeks, and decreased activation of its key receptor, VEGFR2 at 7 days and 4 weeks post-instillation. Together, these data provide insight on long-term pulmonary functional outcomes 4 weeks after ALI and identify angiogenic proteins as possible therapeutic targets following lung injury.

## Introduction

Acute lung injury (ALI) and acute respiratory distress syndrome (ARDS) are characterized by the onset of bilateral lung infiltrates with alveolar injury, hyaline membrane formation, and protein-rich edema resulting in severe hypoxemia^1,2^. Clinically, ARDS is associated with significant morbidity and mortality and has a number of causes including underlying sepsis, bacteria/viral pneumonia, and trauma. It is estimated that amongst patients admitted to intensive care units, up to 15% meet criteria for ARDS, including 23% of ventilated patients^3^. Despite advances in critical care, there is currently no effective medication targeting the underlying pathophysiology of ALI and management remains supportive with a focus on addressing the inciting cause^1^. The high incidence of Coronavirus disease 2019 (COVID-19)-induced ARDS has highlighted the urgent need to better understand the underlying pathophysiology involved in ALI^4,5^.

Lipopolysaccharide (LPS)-induced lung injury is the most commonly utilized murine model of ALI and shares similarities in pathophysiology to human ARDS^6^. While the acute effects of LPS-induced murine ALI are well-characterized, there is a paucity of data on the long-term pulmonary effects of isolated LPS exposure and lung recovery. In one study of isolated nebulized LPS exposure, persistent lung inflammation and remodeling was observed at 5 weeks^7^. Investigation of the long-term effects of ALI in the murine model can improve understanding of persistent symptoms in humans following recovery from ARDS/ALI. Observational studies have shown that over one-third of patients with COVID-19 experience persistent long-term symptoms and patients have impaired pulmonary function following resolution of the acute infection^8–10^.

Lung regeneration through vascular remodeling and angiogenesis may be involved in the recovery from ALI/ARDS^11,12^. For instance, some data suggest that lung recovery may be characterized by dysregulation of angiogenic signaling, including the vascular endothelial growth factor (VEGF) signaling pathway^13^. An improved understanding of these pathways may reveal novel therapeutic targets that could improve pulmonary function after ALI/ARDS.

In this study, we examined metrics of lung recovery in both the short-term (24 h to 7 days) and long-term (4 weeks) following LPS-induced murine lung injury. Metrics included pulmonary function testing (PFT), treadmill exercise tolerance testing (TETT), lung morphometrics, and markers of inflammation. Long-term changes in angiogenic signaling and vascularization were also evaluated. We hypothesized that mice exposed to LPS would demonstrate diminished pulmonary functional outcomes that persist in the long-term and that pro-angiogenic signaling pathways would be affected.

## Results

### Body weight significantly decreased in LPS-treated mice in the short-term

LPS-treated animals lost weight from baseline compared to controls who lost little to no weight at 24 h (− 10.3% vs. − 4.2%, *P* < 0.0001), 4 days (− 14.1% vs. + 0.9%, *P* < 0.0001) and 7 days (− 6.2% vs. − 0.8%, *P* = 0.0009) following LPS instillation (Supplemental Fig. 1A–C). Both groups gained weight at 4 weeks, and there was no significant difference in weight gain between the two groups (8.7% vs. 6.9%, *P* = 0.28; Supplemental Fig. 1D). LPS mice developed clinical signs of inflammation at 24 h and 4 days, including decreased activity and ruffled fur, that was not evident at 7 days and 4 weeks following exposure (data not shown). No animals died as a result of LPS administration.

### LPS-induced lung injury results in long-term changes in pulmonary function testing

**Figure 1:**
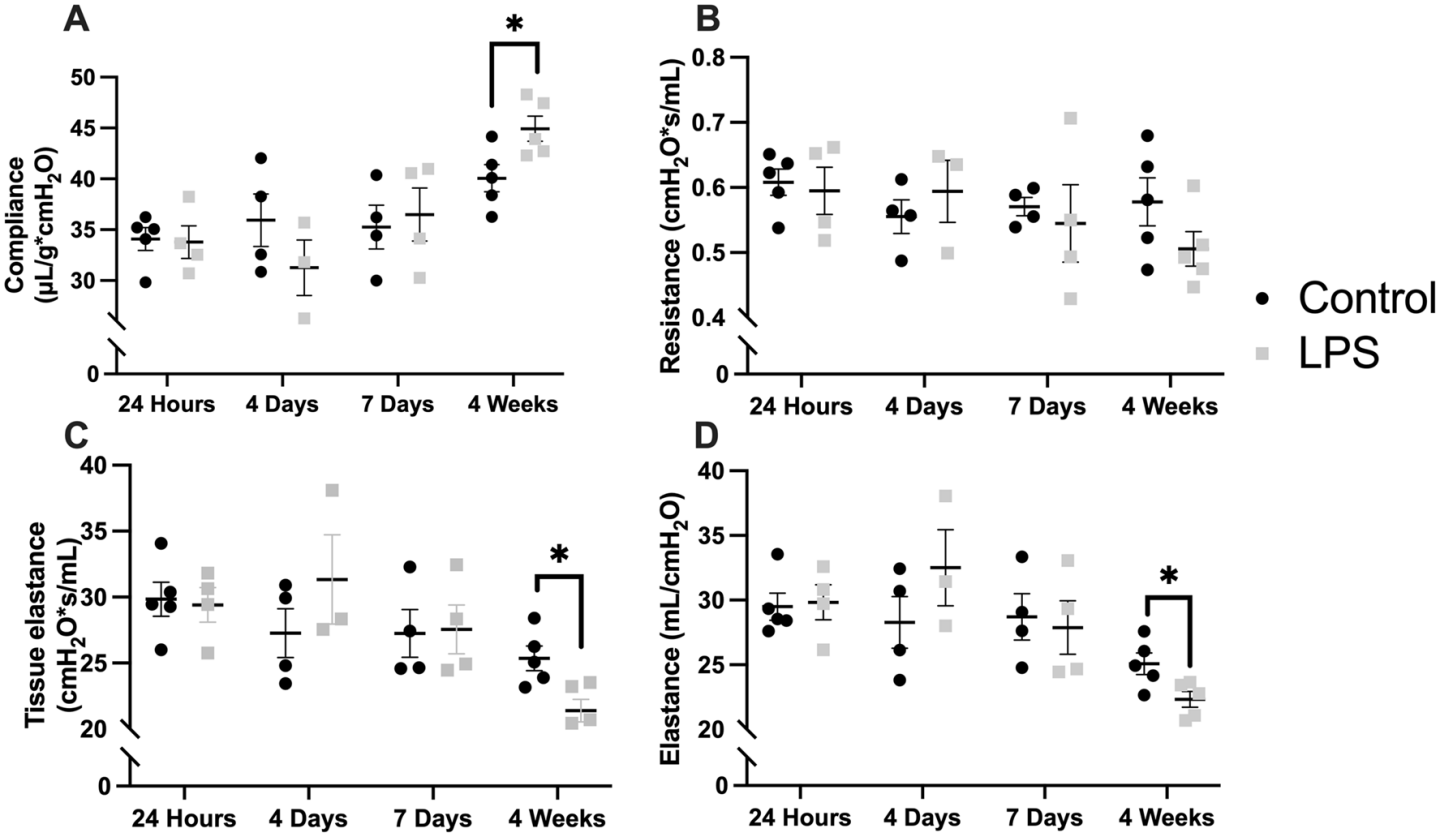
Pulmonary function testing. Lipopolysaccharide (LPS)-treated mice had similar pulmonary compliance compared to control mice at 24 h, 4 days, and 7 days after instillation but significantly increased compliance at the 4 week time point (**A)**. There was no statistically significant difference in lung resistance **(B)** between the two groups at the various points. Tissue elastance **(C)**, and pulmonary elastance **(D)**, measures of lung elastic recoil, were also significantly lower in LPS-treated mice compared to controls at 4 weeks. Compliance was adjusted for mouse body weight. Statistical analysis of the experimental groups at each time point was performed with Student’s t-test. As the experiment is terminal, each time point is represented by a separate cohort. Results are expressed as mean ± SE. *P < 0.05.

Separate cohorts of mice underwent PFT measurements at various time points of euthanasia (Fig. 1). LPS-treated mice had similar compliance compared to control mice at 24 h (33.8 vs. 34.1 μL/cmH_2_O**,** *P* = 0.88), 4 days (31.3 vs. 35.9 μL/cmH_2_O, *P* = 0.27), and 7 days (36.5 vs 35.3 μL/cmH_2_O, *P* = 0.73) following instillation (Fig. 1A). At 4 weeks, pulmonary compliance was significantly increased in the LPS group compared to controls (44.9 vs. 40.1 μL/cmH_2_O, *P* = 0.03). There were no differences in lung resistance at the various time points (Fig. 1B). The increase in compliance at 4 weeks also correlated with decreased lung elastic recoil as measured by the tissue elastance (Fig. 1C) and overall elastance (Fig. 1D). Specifically, the tissue elastance at 4 weeks in LPS-treated mice was 21.4 cmH_2_O*s/mL compared to 25.4 cmH_2_O*s/mL measured in controls (*P* = 0.01). Elastance was also lower in the LPS group at 4 weeks (22.3 vs. 25.1 mL/cmH_2_O, *P* = 0.03). There were no differences in both measures of elastic recoil at the remaining time points (Fig. 1C,D).

### LPS administration produced an acute elevation in inflammatory markers

**Figure 2:**
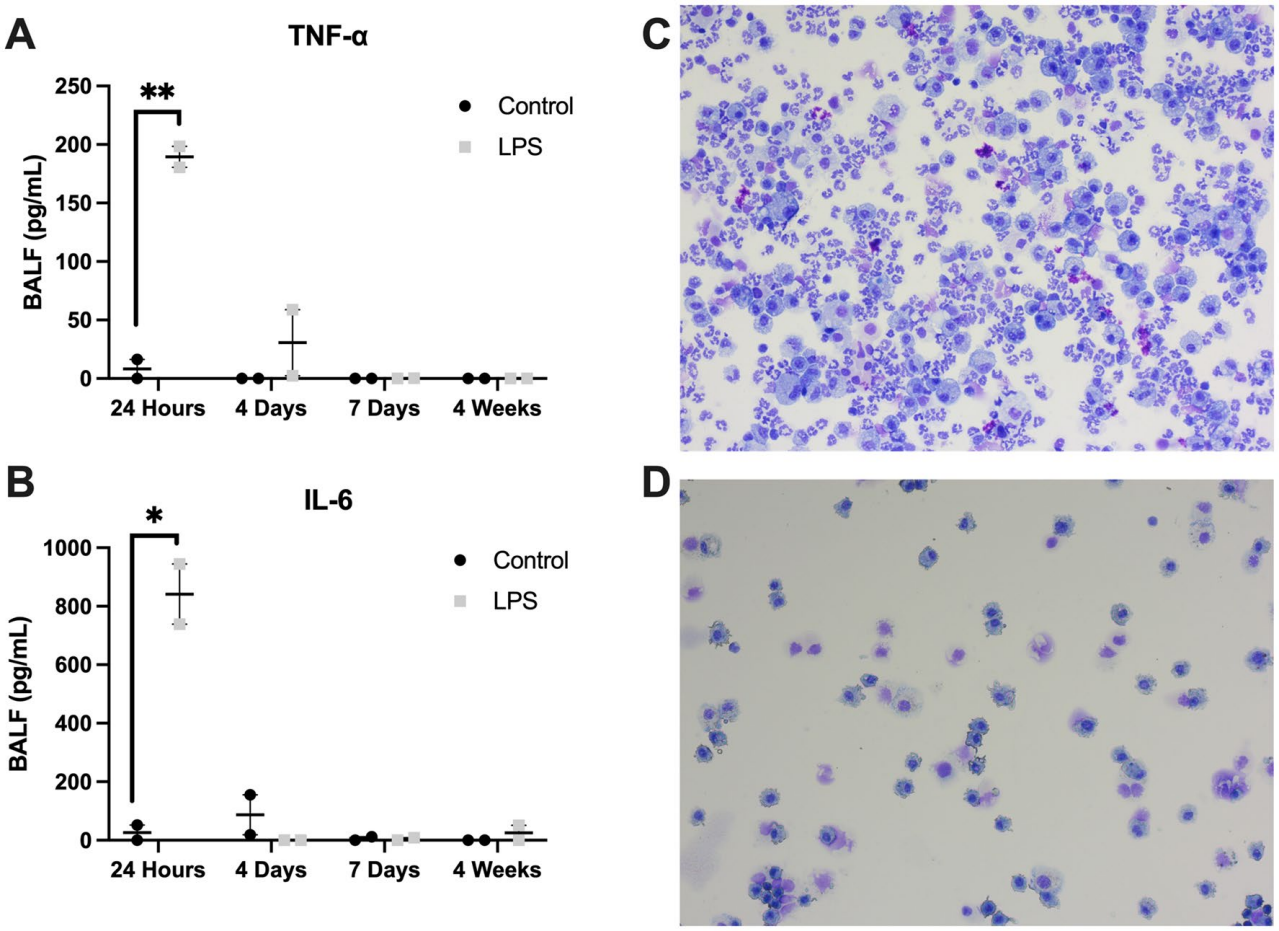
Bronchoalveolar fluid (BALF) analysis. Markers of acute inflammation including Tumor necrosis factor (TNF)-a (**A)** and Interleukin (IL)-6 **(B)** were elevated in the BALF in lipopolysaccharide (LPS)-treated mice compared to controls at 24 h following instillation. There was no significant difference in the levels of TNF-a and IL-6 at 4 days, 7 days, and 4 weeks. Representative slides at 200 × magnification of the BALF cells for LPS-treated mice reveal an inflammatory infiltrate composed primarily of neutrophils at the 4 day time point (**C**), which was not present at 4 weeks (**D**). Each result was performed in biological and technical duplicate. Statistical analysis of the experimental groups at each time-point points was performed with Student’s t-test. Control mice were compared across all four time points based on analysis of variance (ANOVA) and found to have no significant differences in TNF-α or IL-6. Results are expressed as mean ± SE. *P < 0.05; **P < 0.01.

The bronchoalveolar fluid (BALF) was analyzed for white blood cells and markers of acute inflammation. LPS-treated mice at 24 h had significant elevations in both TNF-α (Fig. 2A) and IL-6 (Fig. 2B) compared to controls. Both markers were similar in control and LPS-treated mice at 4 days, 7 days, and 4 weeks. There were no significant differences in the concentration of TNF-α or IL-6 in control mice across all four time points (*P* = 0.48 and *P* = 0.42, respectively).

Representative slides at 200 × magnification of cells isolated from the BALF of LPS-treated mice revealed an inflammatory infiltrate composed primarily of neutrophils at 4 days following instillation (Fig. 2C), which was not present at 4 weeks (Fig. 2D). There were no significant differences observed in the absolute count of the various cells in the control group at 4 days and 4 weeks (Supplemental Fig. 2A, B). Neutrophils were significantly elevated and represented 72.1% of cells in the BALF of LPS-treated mice at 4 days (Supplemental Fig. 2A, C) and just 0.3% of cells at 4 weeks (Supplemental Fig. 2B, C). Monocytes were the predominant cell type in both control (72.1%) and LPS (52.0%) mice at 4 weeks. On average, LPS-treated mice had a higher percentage of lymphocytes than control mice at 4 weeks (Supplemental Fig. 2D), but this was not statistically significant (47.7% vs. 27.1%, *P* = 0.55).

### LPS-induced ALI produces progressive decrease in alveolarization in the long-term

**Figure 3:**
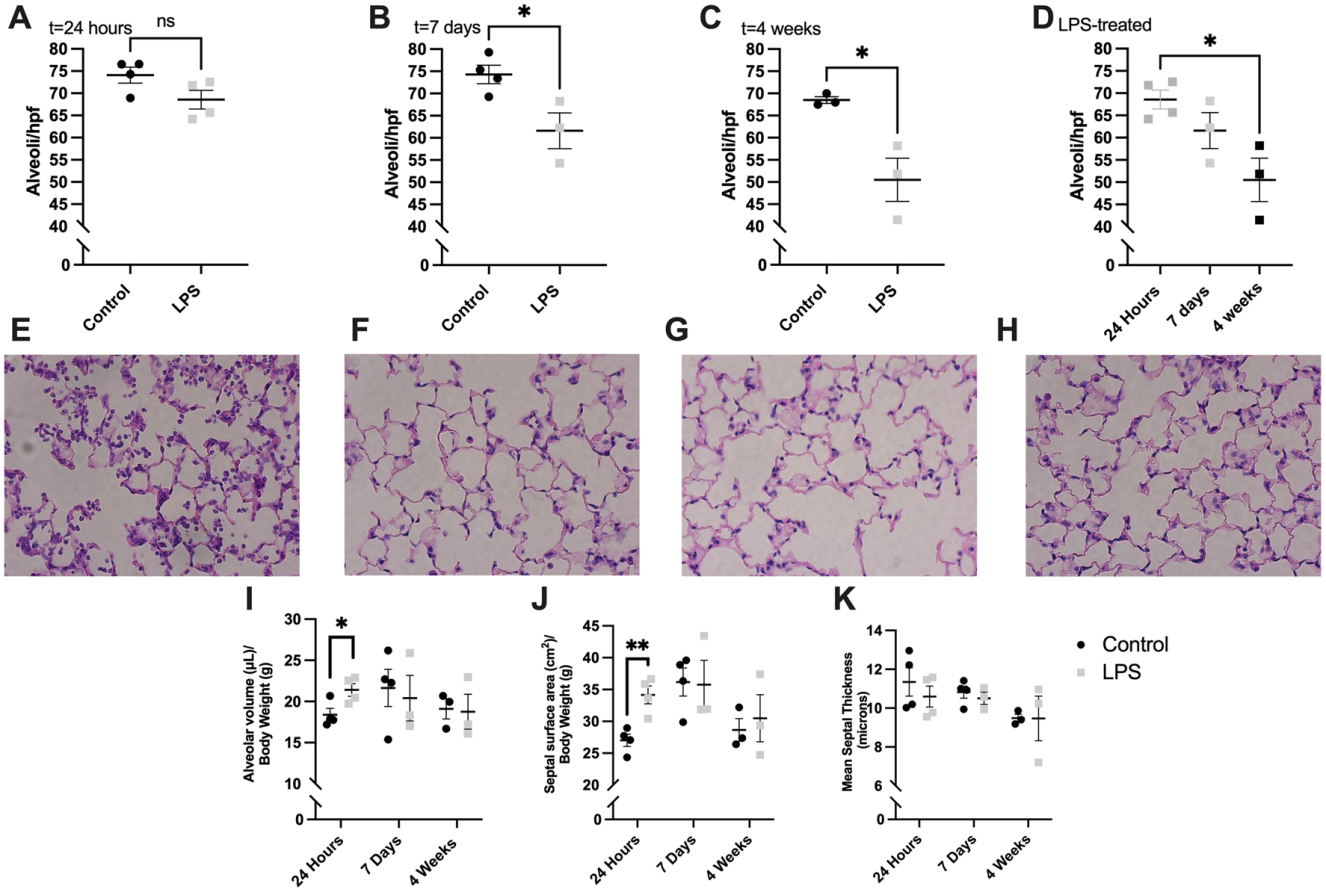
Lung tissue morphometric analysis. Lipopolysaccharide (LPS)-treated mice had similar number of alveoli to controls at 24 h following instillation (**A**). However, LPS-treated mice exhibited a progressive decrease in alveolar counts compared to control mice at 7 days (**B**) and 4 weeks (**C**) after instillation. The lungs of LPS-treated mice at 4 weeks had significantly lower number of alveoli compared to the lungs of LPS-treated mice at 24 h (**D**). Representative micrographs of hematoxylin and eosin-stained lung sections at 400 × magnification demonstrate progressive alveolar simplification in LPS-treated mice from 24 h following instillation (**E**), to 7 days (**F**), and 4 weeks (**G**). Representative micrograph of control lungs at 24 h is provided for reference (**H**). Alveolar volume (**I)** and septal surface area (**J**) were significantly higher in LPS-treated mice compared to controls at 24 h but there were no significant differences at 7 days and 4 weeks. There were no significant differences in mean septal thickness (**K**) at the various time points. Alveolar counts were determined by two independent masked reviewers and averaged. Statistical analysis of the experimental groups at each time point was performed with Student’s t-test. Comparison of the alveolar counts in LPS mice was done using a one-way analysis of variance (ANOVA) with Tukey’s adjustment for multiple comparisons. Results are expressed as mean ± SE. hpf: high power field; ns: nonsignificant; *P < 0.05; **P < 0.01.

Formalin-fixed paraffin-embedded lungs stained with H&E were examined at 400 × magnification. There was no statistical difference in the number of alveoli in LPS-treated mice compared to controls at 24 h post-instillation (Fig. 3A; 68.6 vs. 74.1 alveoli/hpf, *P* = 0.09). The mean number of alveoli was lower in LPS-treated mice at 7 days (Fig. 3B; 61.6 vs. 74.3 alveoli/hpf, *P* = 0.03) and was even lower at 4 weeks (Fig. 3C; 50.5 vs. 68.5 alveoli/hpf, *P* = 0.02). Overall there was a progressive decrease in alveoli across the three time points (Fig. 3D; *P* = 0.02). Representative micrographs obtained at 400 × magnification demonstrated progressive alveolar simplification in the LPS-treated mice from 24 h (Fig. 3E) to 7 days (Fig. 3F), to 4 weeks from instillation (Fig. 3G).

Alveolar volume (Fig. 3I) was higher in LPS-treated mice compared to controls at 24 h (21.4 vs. 19.4 μL/g, *P* = 0.03). Similarly, septal surface area (Fig. 3J) was also increased at 24 h in LPS mice (34.2 vs. 27.0 cm^2^/g, *P* = 0.00572). There were no significant differences in alveolar volume or septal surface area at 7 days and 4 weeks post-instillation. In addition, there were no significant differences observed in mean septal thickness at any of the three time points (Fig. 3K).

### LPS administration resulted in decreased vascularization in the long-term

**Figure 4:**
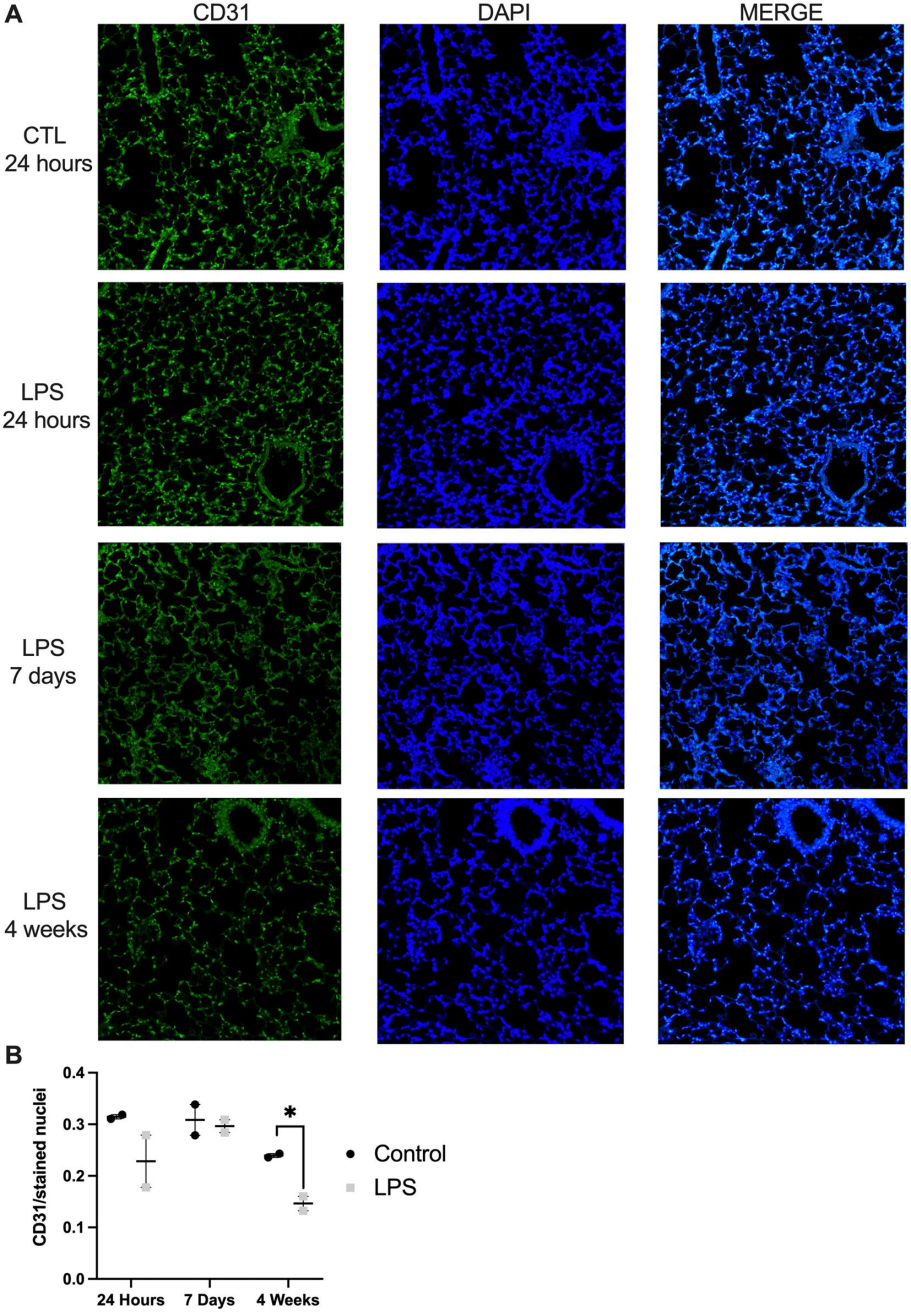
Immunohistochemistry to assess lung vascularization. Representative micrographs at 200 × magnification of co-stained lung tissue for the endothelial cell marker CD31 (green) and nuclear marker DAPI (blue) at 24 h, 7 days, and 4 weeks (**A**). Based on quantification, CD31 significantly decreases at 4 weeks in LPS-treated mice compared to controls (**B**). Statistical analysis of the experimental groups at each time point was performed with Student’s t-test. Results are expressed as mean ± SE. *P < 0.05.

Immunohistochemistry (IHC) was performed to describe the histological basis of observed mechanical and morphometric changes. There were no significant differences in the proportion of type II pneumocytes between LPS-treated mice and controls at 24 h (18.1% vs. 15.1%, *P* = 0.76), 7 days (7.4% vs.5.6%, *P* = 0*.*25*)*, and 4 weeks (17.1% vs. 10.8%, *P* = 0.33). Vascularization was assessed using the CD31 endothelial cell marker (Fig. 4) as previously described^14^. There were no significant differences in CD31 staining between LPS-treated mice and controls at 24 h and 7 days. However, at 4 weeks, LPS-treated mice demonstrated a significant reduction in CD31 staining compared to controls (Fig. 4B; 0.15 vs. 0.24 antibody/stained nuclei, *P* = 0.02).

Type I (COL1A1) and type IV collagen (COL4A1) content were also assessed on IHC (Supplemental Fig. 3). There was no statistically significant difference between groups in type I collagen content at the various time points (Supplemental Fig. 3B). Type IV collagen was lower in LPS-treated mice compared to controls (Supplemental Fig. 3C) at 4 weeks (0.6 vs. 1.2 antibody/stained nuclei, *P* = 0.02) but there was no statistical difference at the remaining time points. The amount of type IV collagen increased in control mice between 24 h and 4 weeks (0.6 vs. 1.2 antibody/stained nuclei, *P* = 0.01).

### LPS-treated mice had reduced exercise tolerance compared to control mice at up to 4 weeks following instillation

**Figure 5:**
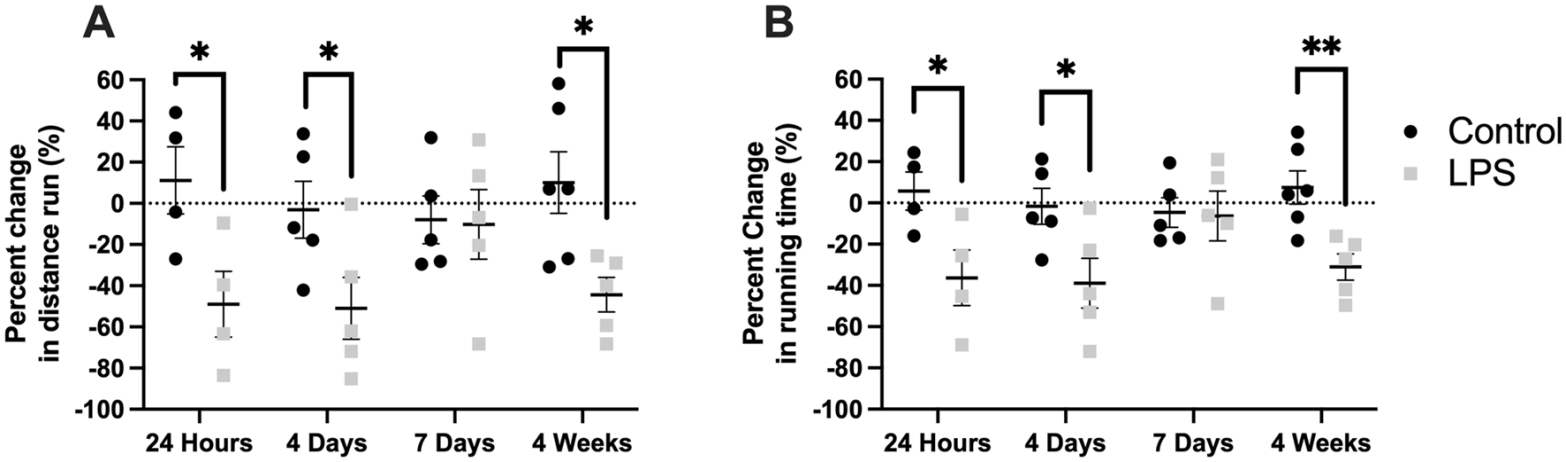
Treadmill exercise tolerance testing (TETT). Mice underwent baseline exercise testing prior to lipopolysaccharide (LPS) exposure followed by a single repeat test at the respective time point of interest for the group. Exercise tolerance was significantly decreased in LPS-treated mice at 24 h, 4 days and 4 weeks following instillation, as measured by the percent change from baseline in distance run (**A**), and the percent change from baseline in time spent running (**B**). Statistical analysis of the experimental groups at each time point was performed with Student’s t-test. Results are expressed as mean ± SE. *P < 0.05; **P < 0.01.

Mice underwent baseline exercise tolerance testing prior to LPS exposure followed by repeat testing at various time points. Exercise tolerance was measured in terms of the percent change in distance run (Fig. 5A) and time spent running (Fig. 5B) from baseline; thus, each mouse effectively served as its own control. LPS and control mice had similar baseline exercise performance in terms of distance run (*P* = 0.30) and time spent running (*P* = 0.29) (data not shown). LPS-treated mice had significant percent reduction in the distance run and running time compared to controls at 24 h (distance: − 49.0% vs. 11.2%, *P* = 0.04; time: − 36.3% vs. 5.8%, *P* = 0.04) and 4 days (distance: − 51.0% vs. − 3.1%, *P* = 0.047; time: − 38.9% vs. − 1.7%, *P* = 0.04). At the 7 day time point, LPS mice performed similar to control mice (distance: − 10.2% vs. − 8.0%, *P* = 0.91; time: − 6.3% vs. − 4.6%, *P* = 0.90). However, LPS-treated mice demonstrated reduced exercise tolerance compared to controls at the later 4 week time point (distance: − 44.4% vs. 10.1%, *P* = 0.01; time: − 31.0% vs. 7.5%, *P* = 0.00550).

### Exposure to LPS results in downregulation of VEGF and downstream signaling pathways

**Figure 6:**
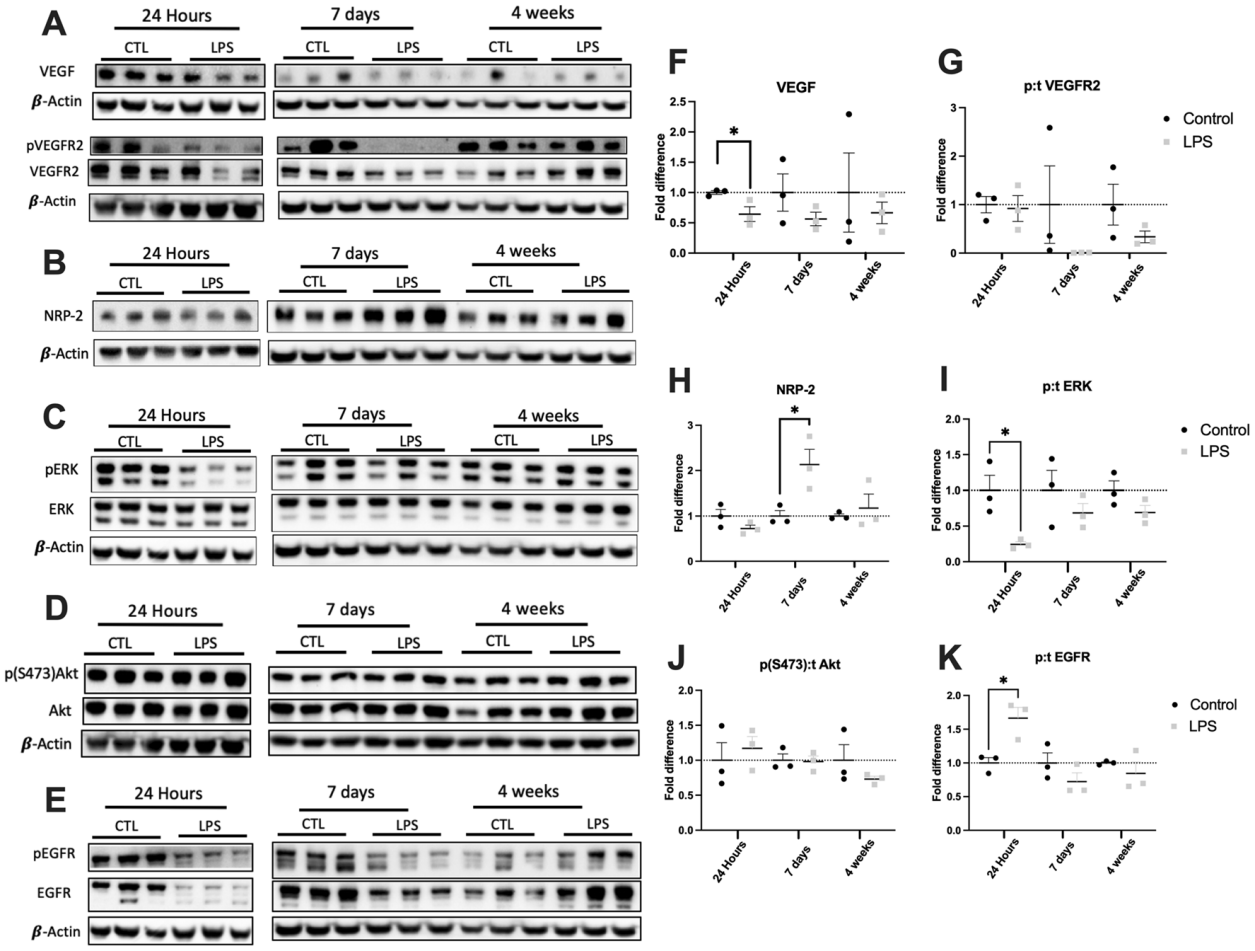
Lung tissue immunoblots. Pulmonary VEGF expression was lower in lipopolysaccharide (LPS)-treated mice compared to controls (CTL) at all time points (**A, F**) but was statistically significant only at 24 h after instillation (**F**). Activation of VEGFR2 (phosphorylated/total receptor VEGFR2) was also lower in LPS-treated mice at 7 days and 4 weeks following instillation (**G**). LPS exposure significantly upregulated expression of the co-receptor neuropilin 2 (NRP-2) at 7 days (**B, H**). Activation of the downstream proliferation marker ERK (phosphorylated/total receptor ERK) was significantly lower at 24 h after LPS (**C, I**) while there were no differences in AKT-S473 activation at the various time points (**D, J**). Activation of an alternative pathway involving the epithelial growth factor receptor (phosphorylated/total EGFR) was significantly increased at 24 h following LPS instillation (**E, K**). Uncropped blots are provided in Supplemental Information Files 1–4. The β-actin for each membrane is displayed below the corresponding antibodies and was used to normalize the expression patterns. Each lane represents a sample from a different mouse. Statistical analysis of protein expression at each time point was performed with Student’s t-test. Results are expressed as mean ± SE. *P < 0.05.

Lung tissue protein was analyzed to determine the role of angiogenic signaling pathways in long-term recovery from ALI (Fig. 6). Pulmonary VEGF expression was lower in LPS-treated mice compared to controls at all time points (Fig. 6A,F) but this was statistically significant only at 24 h from LPS instillation (0.64-fold, *P* = 0.047). The phosphorylated active form of VEGF receptor 2 (pVEGFR2) was undetectable at 7 days from exposure and VEGFR2 activation remained decreased at 4 weeks as demonstrated by the decreased ratio of phosphorylated to total VEGFR2 (Fig. 6G). Neuropilin-2 (NRP-2) is a co-receptor for VEGF and is thought to mediate VEGFR2 signaling^15^. NRP-2 was found to be upregulated in LPS mice compared to controls at 7 days following instillation (Fig. 6B,H; 2.13-fold, *P* = 0.03).

These changes correlated with a decrease in the activation of the downstream effector pathway involving MAPK/ERK (Fig. 6C,I) at 24 h following instillation (0.24-fold, *P* = 0.02). An additional effector pathway involving AKT was not affected by LPS instillation at the various time points (Fig. 6D,J). Activation of epithelial growth factor receptor (EGFR) was significantly higher in LPS-treated mice (Fig. 6E,K) as demonstrated by an increased ratio of phosphorylated to total EGFR at 24 h post-instillation (1.67-fold, *P* = 0.02).

## Discussion

Animal models of ALI/ARDS have the potential to offer valuable insight into the underlying complex pathophysiology as it relates to human disease. LPS-induced murine lung injury is one of the most widely studied models of ALI/ARDS and has been used to expand our understanding of underlying mechanisms and drug therapeutics^16^. However, most studies in the literature have focused on the acute phase of ALI. The current COVID-19 pandemic has highlighted the potentially severe lasting effects following recovery from ARDS and the lack of targeted therapeutics^8,9^.

In this study, we sought to investigate the long-term functional and structural changes of LPS-induced lung injury as it relates to lung regeneration. We have demonstrated that LPS injury in a murine model results in changes in pulmonary function, alveolarization, exercise tolerance, and angiogenic signaling at up to 4 weeks following exposure. Our model displayed the relevant features of ALI including elevated inflammatory markers in the BALF (Fig. 2A,B), an intense inflammatory infiltrate composed primarily of neutrophils (Fig. 2C, Supplemental Fig. 2), and histologic evidence of tissue injury (Fig. 3, Supplemental Fig. 3). LPS-treated mice also developed profound weight loss compared to control mice which normalized by 4 weeks post-instillation (Supplemental Fig. 1). These features are widely accepted as the most relevant and important in the murine LPS-induced lung injury model^4,6^.

Pulmonary function testing failed to demonstrate significant changes in pulmonary compliance, resistance, or elastance at up to 7 days following LPS instillation (Fig. 1A–D). In one study, LPS-treated C57BL/6J mice developed increased resistance and elastance at 1 and 4 days with no significant difference at 10 days after exposure^17^. However, the authors noted that the increases were mild in comparison to what is observed in ARDS patients^17^. This could explain why changes in PFTs were not observed at the early time points in our study and may be a limitation of the LPS murine model compared to human ARDS.

While changes in lung compliance are an important aspect of ARDS, there are limited reports on this and other pulmonary mechanics related to hypoxemia^18^. Interestingly, we observed that compliance significantly increased at the 4 week time point in LPS-treated mice (44.9 vs. 40.1 μL/cmH_2_O, *P* = 0.03). This mechanical change correlated with decreased elastic recoil at 4 weeks (Fig. 1C,D) but did not correlate with changes in the proportion of type II pneumocytes which are responsible for surfactant production and lung homeostasis^19^. Prior studies that have examined the effects of repeated LPS exposure have demonstrated changes in parenchymal architecture that are consistent with emphysema^20,21^. However, no study to our knowledge has shown changes in pulmonary compliance and elastance at 4 weeks after a single dose of LPS in an ALI murine model. The data presented in this study support the notion that ALI can result in the development of long-term diminished pulmonary function and chronic lung conditions such as pulmonary fibrosis and emphysema. These findings are consistent with recent clinical studies demonstrating a high prevalence of diminished pulmonary function in mechanically ventilated survivors of COVID-19 ARDS at 3 months following hospital discharge^22^. Our findings suggest that the murine model of LPS-induced ALI can be valuable in investigating the pathophysiology and therapeutics of such long-term sequalae.

The extent of changes in lung architecture due to LPS exposure in this model was also determined (Fig. 3). LPS-treated mice had a progressive decrease in the number of alveoli compared to control mice starting from 7 days post-instillation. The number of alveoli decreased from 68.6 alveoli/hpf at 24 h to just 50.5 alveoli/hpf at 4 weeks (*P* = 0.01). Representative slides of H&E-stained lung sections demonstrated the LPS-induced progressive alveolar simplification over time. There were no significant differences observed in other morphometric parameters (alveolar volume, septal surface area, septal thickness) at 7 days and 4 weeks. The decrease in the number of alveoli by 4 weeks is consistent with the observed changes in pulmonary compliance and loss of elastic recoil at that time point. In one study, authors examined the late pulmonary effects of single-dose nebulized LPS and found that alveolar volume and septal thickness mildly increased at 5 weeks from exposure in contrast to our findings^7^. Differences in LPS administration as well as lack of time point specific control groups in that study could explain these discrepancies.

The significant decrease in the number of alveoli at 4 weeks in LPS-treated mice correlated with decreased vascularization at that time point as assessed by staining for the CD31 endothelial cell marker (Fig. 4). This finding is not surprising given the known association between the pulmonary vascular bed and alveolarization during development and pulmonary growth^23^. However, no prior study to our knowledge has previously demonstrated these changes in the long-term ALI model.

Architectural changes were also characterized by immunostaining for type I and type IV collagen (Supplemental Fig. 3). There was no significant increase in type I collagen in the LPS-treated mice, in contrast to other reports in the literature^7,20^. However, type I collagen may appear to be redistributed over time from the alveolar capillary membrane to the nonparenchymal tissue and bronchial walls (Supplemental Fig. 3A). This effect was most pronounced at the 4 week time point and was previously demonstrated at up to 10 days following acute lung injury in another study^24^. Interestingly, type IV collagen was lower in LPS-treated mice compared to controls at 4 weeks following exposure (Supplemental Fig. 3C). Type IV collagen is the main component of the epithelial and endothelial basement membranes and contributes to the strength of the blood-gas barrier^25,26^. Recent data suggests that type IV collagen plays a fundamental role in coordinating alveolar morphogenesis and pulmonary angiogenesis and increases in amount over the course of gestation^27^. Given these relationships, the observed changes may reflect a maladaptive response to the inflammatory insult in ALI that may be related to the observed decreases in alveolarization and vascularization. Of note, type IV collagen increased in control mice between 24 h and 4 weeks. This may reflect age-dependent changes in pulmonary architecture and highlights the importance of including control groups at each time point for comparison.

The change in pulmonary mechanics and lung architecture at 4 weeks correlated with decreased exercise tolerance compared to controls (Fig. 5). The use of exercise tolerance testing as a functional measurement of lung capacity is well established^28–30^. These results suggest that isolated LPS exposure contributes to impaired long-term functional capacity. These secondary consequences of ALI/ARDS have recently become increasingly recognized in the setting of diminished pulmonary function following recovery from COVID-19 ARDS^10^. Of note, LPS-treated mice also performed worse than controls at 24 h and 4 days but had similar exercise tolerance at 7 days from instillation. In light of normal PFTs and alveolar counts at these earlier time points, the poor exercise performance may be due to the systemic effects of the inflammatory response to LPS. This is supported by the increased levels of inflammatory markers (TNF-a and IL-6), neutrophils, and substantial weight loss observed at 24 h and 4 days from exposure. However, at 7 days, these inflammatory markers were normal and the mice had no further clinical signs of inflammation, which could explain the improved exercise tolerance at that time point.

Lung protein analysis revealed decreased VEGF expression in LPS-treated mice at all time points with concomitant decreased activation of VEGFR2 at 7 days and 4 weeks following instillation (Fig. 6A,F,G). The decrease in VEGFR2 activity was observed despite upregulation of NRP-2, an important cofactor in VEGF signaling^15^. These changes correlated with decreased activation of the downstream proliferative MAPK/ERK effector pathway but not the AKT pathway (Fig. 6C,D,I,J). Activation of EGFR was significantly increased at 24 h following LPS exposure (Fig. 6E,K), which is consistent with prior studies^31^. Increased EGFR activity may worsen ALI, as inhibition of EGFR by erlotinib alleviated LPS-induced ALI^31^.

Prior studies suggest that VEGF may have a pathological role in ALI/ARDS in the short-term by contributing to increased permeability of the capillary membrane leading to noncardiogenic pulmonary edema^32^. However, other studies have found that VEGF may function through autocrine signaling as a pneumotrophic factor in the lung, facilitating recovery from lung injury in the long-term^11,33–36^. These contrasting results are explained by considering that VEGF may have differential effects in ALI depending on the location of action: in the capillaries (increases permeability) versus alveolar compartment (acts as a pneumotrophic factor)^12^. However, based on our data, the long-term decrease in VEGF activity may reflect a maladaptive response to LPS injury and hinder recovery from ALI, especially considering the observed decrease in vascularization at 4 weeks. Prior work in our group has shown that exogenous VEGF administration increases alveolarization in a compensatory lung growth model^37,38^. Considering these prior studies, the concomitant decrease in VEGF signaling and the diminished vascularization and alveolarization observed at 4 weeks from LPS instillation offers a potential therapeutic target that warrants further investigation at the pre-clinical level. Future work should focus on studying the effects of exogenous administration of VEGF and its potential effects on pulmonary function and alveolarization in the ALI model.

There are several key differences between ARDS in humans and mice that limit clinical translation of the LPS-induced lung injury model. While LPS injury recapitulates part of the pathophysiology observed in ARDS, it has important limitations in modeling the disease process. For example, while human ARDS is characterized clinically by initial reduction in compliance, no change in pulmonary compliance was observed in the short-term. The addition of ventilation-induced lung injury to LPS instillation in this model may offer added benefits as suggested by prior studies^39^. Despite these inherent limitations, the LPS-induced murine model of ALI is well established in the literature and its reproducibility allows for valuable insights into human disease^4^. This study has additional important limitations. Blood oxygenation was not measured, although hypoxemia is important in clinical ARDS. In addition, only male mice were utilized in this study and sex differences in response to LPS instillation cannot be excluded. Furthermore, while we do establish a correlation between diminished pulmonary function and reduction in VEGFR2 activation in the long term, we do not provide direct evidence that VEGFR2 activation will improve pulmonary function. Finally, only specific snapshots in time are captured in this study. This is an important limitation in the description of a continuous physiological process.

In conclusion, LPS-induced murine lung injury results in long-term functional and structural changes, as demonstrated by the increased pulmonary compliance and impaired exercise tolerance that persist at 4 weeks following injury. These correlated with changes in pulmonary architecture including diminished alveolarization and vascularization. A decrease in pro-angiogenic VEGF signaling correlated with these findings. Together, these data provide a first look at long-term pulmonary functional outcomes in this model and identify angiogenic proteins as possible therapeutic targets in acute lung injury.

## Methods

### Lung injury animal model

Experiments were conducted with 8- to 10-week-old C57BL/6J male mice (Jackson Laboratories, Bar Harbor, ME) weighing approximately 25 g. All animal experiments were carried out according to the National Institutes of Health Guide for the Care and Use of Laboratory Animals and approved by the Institutional Animal Care and Use Committee (Boston Children’s Hospital) and in compliance with the ARRIVE guidelines.

The mice were anesthetized with ketamine (80–100 mg/kg) and xylazine (5–10 mg/kg) via intraperitoneal injection. A 22-gauge catheter (Critikon, Tampa, FL) was inserted into the trachea under direct visualization. Mice were randomized to receive either 75 μL LPS (from *Escherichia coli* O111:BA-Sigma Aldrich, Allentown, PA) at a concentration of 1 μg/μL or 75 μL phosphate buffered saline (PBS) as vehicle control. Following administration, animals were recovered on a heating pad and observed for signs of respiratory distress. Mice were sacrificed at the following time points: 24 h, 4 days, 7 days, and 4 weeks.

### Pulmonary function testing

At the time points of interest, mice were anesthetized with ketamine (80–100 mg/kg) and xylazine (5–10 mg/kg). The trachea was exposed and a tracheostomy was performed with a 20-gauge hollow needle. The mice were then paralyzed with pancuronium (0.8 mg/kg) and connected to the Flexivent ® system (SCIREQ, Montreal, Canada) for PFT measurements as previously described^29^. This system utilizes a forced oscillation maneuver and single compartment model to determine pulmonary compliance (Cp), resistance (Rrs), tissue elastance (H), and elastance (Ers). This procedure is terminal as mice are euthanized during degassing via tracheal occlusion.

### Bronchoalveolar lavage

Following PFT measurements, bronchoalveolar lavage (BAL) was performed with three serial instillations of 0.8 mL of sterile PBS through the tracheostomy. Blood samples were obtained through IVC puncture and collected in EDTA tubes. BAL samples were centrifuged at 800×*g* at 4 °C for 10 min while plasma was separated by centrifugation at 2000×*g* for 20 min. The BAL and plasma supernatant were flash frozen in liquid nitrogen and saved at − 80 °C for further analysis.

The BAL cell pellet was suspended in 100 μL sterile PBS and 1 mL of RBC lysis solution (Miltenyi Biotec, Auburn, CA). The sample was incubated for 10 min at room temperature and then centrifuged at 300×*g* for 5 min. The supernatant was discarded and the cells were resuspended in 100 μL sterile PBS and placed on a cytospin slide. Total and differential counts of BAL fluid (BALF) were determined using the May-Grunwald-Giemsa stain (300 cells per animal) as previously described^7^. Counts were performed in biological duplicates.

### Enzyme-linked immunosorbent assay (ELISA)

The cell-free BAL supernatant was collected at the various time points as described above. The levels of the inflammatory cytokines interleukin-6 (IL-6) and tumor necrosis factor-α (TNF-α) were determined by enzyme-linked immunosorbent assay (ELISA) using specific antibodies and standards according to the manufacturer’s instructions (R&D Systems, Minneapolis, MN). Samples were prepared and analyzed in technical duplicates.

### Organ harvest and morphometric analysis

The lungs of the mice that underwent PFT measurements were removed and inflated with 10% formalin at 35 cmH_2_O. Inflated lung specimens were formalin-fixed for 24 h at 4 °C and transferred to 70% ethanol. Specimens were paraffin embedded for histologic analysis. Lung sections for each experimental group at 24 h, 7 days, and 4 weeks were stained with hematoxylin and eosin (H&E) for quantitative microscopy (N = 3–4 per group). Briefly, 17 lung fields at 200 × magnification per section were selected via systematic uniform random sampling^29^. For each field, a 42-point lattice with grid line was used to perform quantitative microscopy based on the principles of lung stereology^40,41^. This technique allowed for measurement of alveolar volume, septal surface area, and mean septal thickness. Volume and area measurements were normalized to mouse body weight. Additionally, 10 random sections at 400 × magnification were selected from each specimen and the number of alveolar units were counted. All, All measurements were done by two independent masked observers (TIH, MMJ) and averaged.

### Immunohistochemistry

Paraffin-embedded lung sections (N = 2–3 per group) from three time points (24 h, 7 days, 4 weeks) were assessed for type I collagen (COL1A1), type IV collagen (COL4A1), CD31 (endothelial marker), and proSPC (type II pneumocyte marker) expression using immunofluorescence as previously described^30,37^. After deparaffinization with xylene, sections were progressively rehydrated in decreasing concentrations of ethanol, followed by PBS. Epitopes were retrieved by incubating in a citrate-based unmasking solution (Vector Laboratories, Burlingame, CA) at 120 °C in a pressurized chamber (Decloaking Chamber, Biocare Medical, Pacheco, CA). Sections were then permeabilized with 0.05% Tween-20 in phosphate-buffered saline (PBST) for 5 min, followed by incubation in TNB blocking buffer (PerkinElmer Inc, Boston, MA) for 1 h. The slides were then incubated with primary antibodies anti-COL1A1, anti-CD31 (Cell Signaling Technology, Danvers, MA) or anti-COL4A1, and anti-proSPC (Abcam, Cambridge, UK) at 4 °C overnight. Following overnight incubation, slides were washed with PBST and incubated with Alexa-Fluor-conjugated donkey anti-rabbit secondary antibodies (Invitrogen, Carlsbad, CA) for 1 h at room temperature. Sections were counterstained and mounted using Fluoroshield™ with DAPI (Sigma-Aldrich, St. Louis, MO).

The stained sections were examined with confocal microscopy (LSM 800, Zeiss, Jena, Germany) at 200 × magnification. For each specimen, random 20-tiled high-power fields (HPF) spanning the entire lung were captured for analysis. ImageJ v1.53a (National Institutes of Health, Bethesda, MD) was used to quantify.

### Treadmill exercise tolerance testing

A separate cohort of mice from each group was assigned to undergo treadmill exercise tolerance testing (TETT). TETT was used as a metric of pulmonary functional outcome^29^. Mice were individually placed on a stationary treadmill with an attached shock grid (Exer 3/6 Treadmill, Columbus Instruments, Columbus, OH) and acclimated to the apparatus for 5 min at 6 m/min. Following acclimation, mice were exercised on the treadmill, preset to accelerate at a rate of 2 m/min, until exhaustion, defined as remaining on the shock grid for more than 5 s.

TETT was performed two days prior to LPS exposure, and again at 24 h, 4 days, 7 days, and 4 weeks in separate cohorts of mice at each time point. Distance run and time spent running were compared in each mouse pre- and post-LPS, and reported as a percent change compared to baseline values, in order to account for behavioral and intrinsic physiologic differences between mice. Following TETT, the exercised mice were euthanized and samples of both lungs were harvested and flash frozen for molecular analyses as described above.

### Western immunoblot

Approximately 40 μg of lung tissue from three time points (24 h, 7 days, 4 weeks) were suspended in radioimmunoprecipitation assay (RIPA) buffer (Boston Bio Products, Ashland, MA) containing protease and phosphatase inhibitors (Thermo Fischer Scientific, Waltham, MA). Samples were homogenized and centrifuged for 10 min at 12,000 rpm and 4 °C. The supernatant was collected and the protein concentration was determined using the Bradford assay (Thermo Fischer Scientific, Waltham, MA). Protein samples were separated on SDS 4–10% PAGE and transferred to polyvinyl difluoride (PVDF) membranes (Merck Millipore, Darmstadt, Germany). Membranes were blocked in 5% nonfat milk in tris-buffered saline with Tween 20 (TBST) for one hour and incubated in 1:500 to 1:1000 dilution of primary antibodies in 5% nonfat milk-TBST at 4 °C overnight. Primary antibodies included anti-p-Y1175-VEGFR2, -VEGFR2, -NRP2, -pT202/Y204-ERK, -ERK, -p-S473-AKT, AKT, -p-Y1068-EGFR, -EGFR (Cell Signaling Technology, Danvers, MA), and anti-VEGF_120/164_ (R&D Systems, Minneapolis, MN). After washing with TBST, membranes were incubated with horseradish peroxidase (HRP)-conjugated secondary (anti-rabbit or anti-goat) antibody (R&D Systems, Minneapolis, MN) for one hour at room temperature at 1:2000 dilution in 5% nonfat milk-TBST. Blots were normalized by probing with HRP-conjugated β-actin antibody (Sigma-Aldrich, St. Louis, MO). Immunoblots were developed using enhanced chemiluminescence reagents (Bio-Rad, Hercules, CA) on a ChemiDoc Touch System Imager (Bio-Rad, Hercules, CA). Signals were quantified with Image Lab Software v6.1.0 (Bio-Rad, Hercules, CA).

### Statistical analysis

Comparison of exercise test results, pulmonary function tests, total lung volume, and morphometric parameters between control and experimental groups was performed using Student’s t-tests at the various euthanasia time points. Comparisons of the percentages and numbers of cells in the BALF was done using a two-way analysis of variance (ANOVA) model with interaction for group and cell type with Sidak adjustment for multiple comparisons. Results are expressed as mean ± standard error (SE). For all analyses, *P* < 0.05 was considered statistically significant. All analyses were performed on GraphPad Prism v8 (La Jolla, CA).

## Supplementary Information

Supplementary Information 1.

Supplementary Information 2.

Supplementary Information 3.

Supplementary Information 4.

## Data Availability

The datasets generated and/or analyzed during the current study are available from the corresponding author (MP) on reasonable request.

## Abbreviations

ALI: Acute lung injury
ARDS: Acute Respiratory distress syndrome
BAL: Bronchoalveolar lavage
BALF: Bronchoalveolar lavage fluid
COL1A1: Type I collagen
COL4A1: Type IV collagen
EDTA: Ethylenediaminetetraacetic acid
EGFR: Epidermal growth factor receptor
ELISA: Enzyme-linked immunosorbent assay
HPF: High power field
HRP: Horseradish peroxidase
H&E: Hematoxylin and eosin
IHC: Immunohistochemistry
IL-6: Interleukin 6
LPS: Lipopolysaccharide
MAPK/ERK: Mitogen-activated protein kinase/extracellular signal-regulated kinase
NRP-2: Neuropilin-2
PBS: Phosphate-buffered saline
PBST: Phosphate-buffered saline with 0.05% Tween-20
PFT: Pulmonary function testing
PVDF: Polyvinyl difluoride
pEGFR: Phosphorylated epithelial growth factor receptor
pVEGFR2: Phosphorylated vascular endothelial growth factor 2
RBC: Red blood cell
RIPA: Radioimmunoprecipitation assay
TBST: Tris-buffered saline with Tween 20
TETT: Treadmill exercise tolerance testing
TNF-a: Tumor necrosis factor alpha
VEGF: Vascular endothelial growth factor
