# Supplementary figures and images for: Lipopolysaccharide-induced murine lung injury results in long-term pulmonary changes and downregulation of angiogenic pathways
Source: Sci Rep. 2022 Jun 17;12:10245. doi: 10.1038/s41598-022-14618-8 (PMC9205148; doi:10.1038/s41598-022-14618-8)

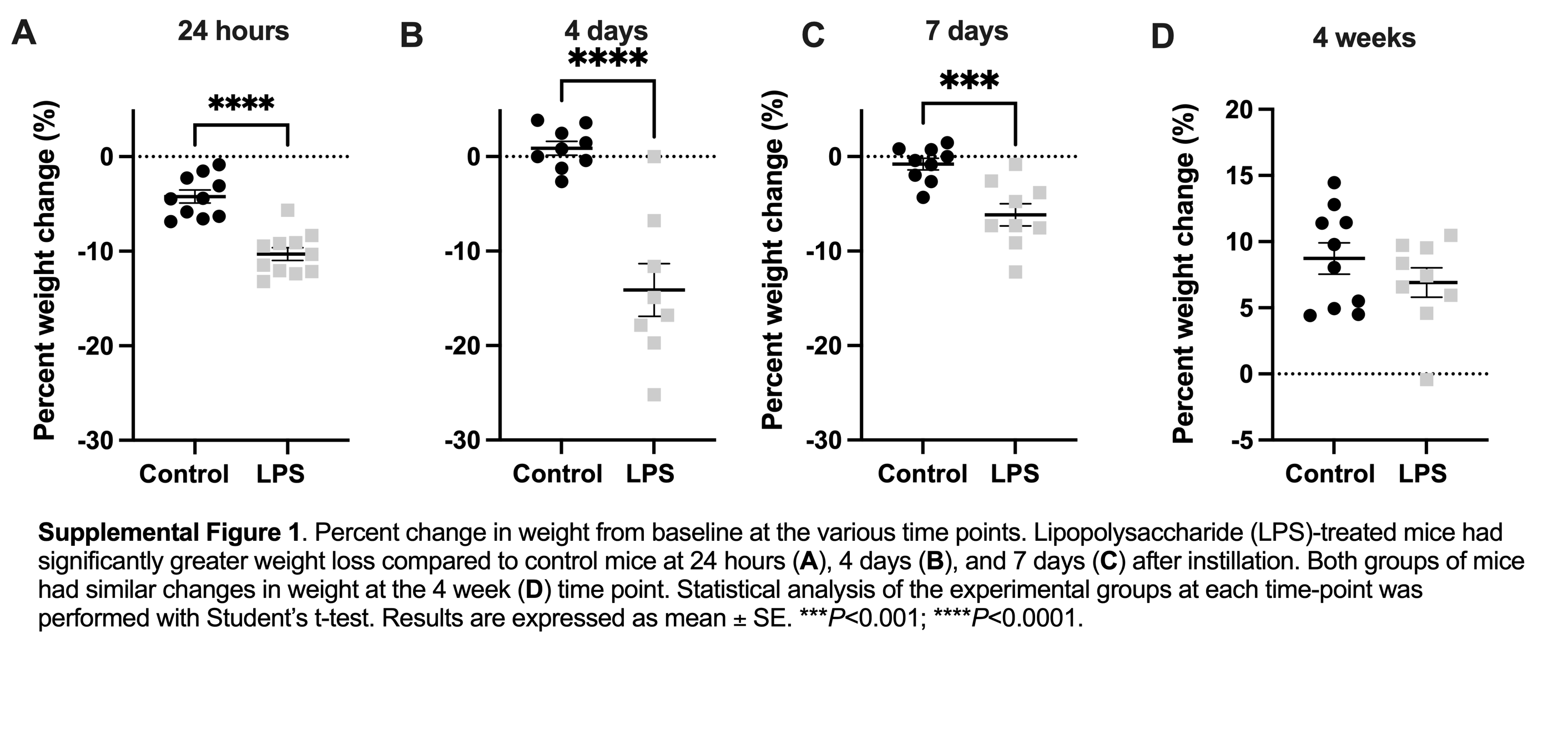

Supplement: Supplementary file 1 — Supplementary Information 1. [file 41598_2022_14618_MOESM1_ESM.tiff]

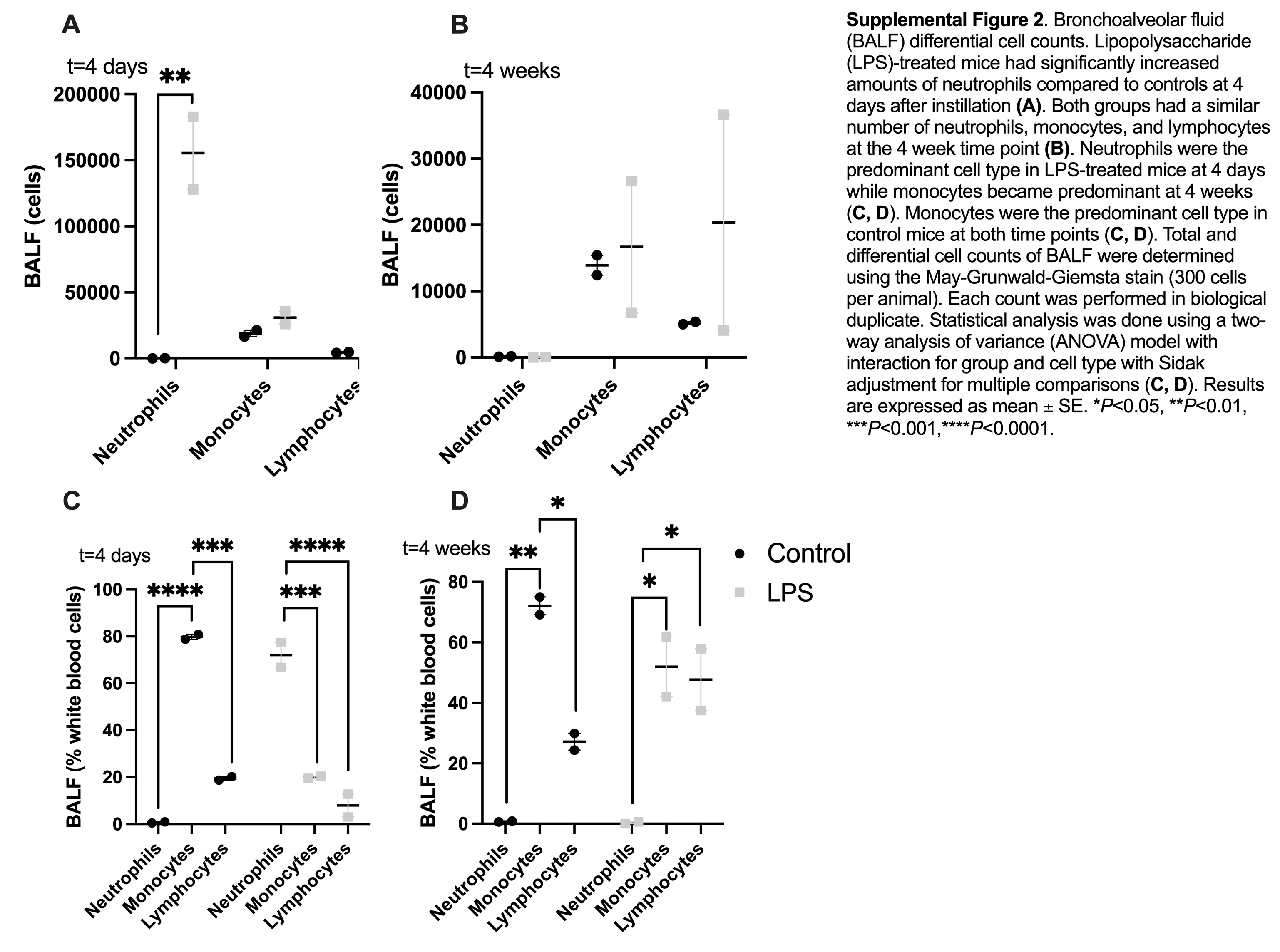

Supplement: Supplementary file 2 — Supplementary Information 2. [file 41598_2022_14618_MOESM2_ESM.tiff]

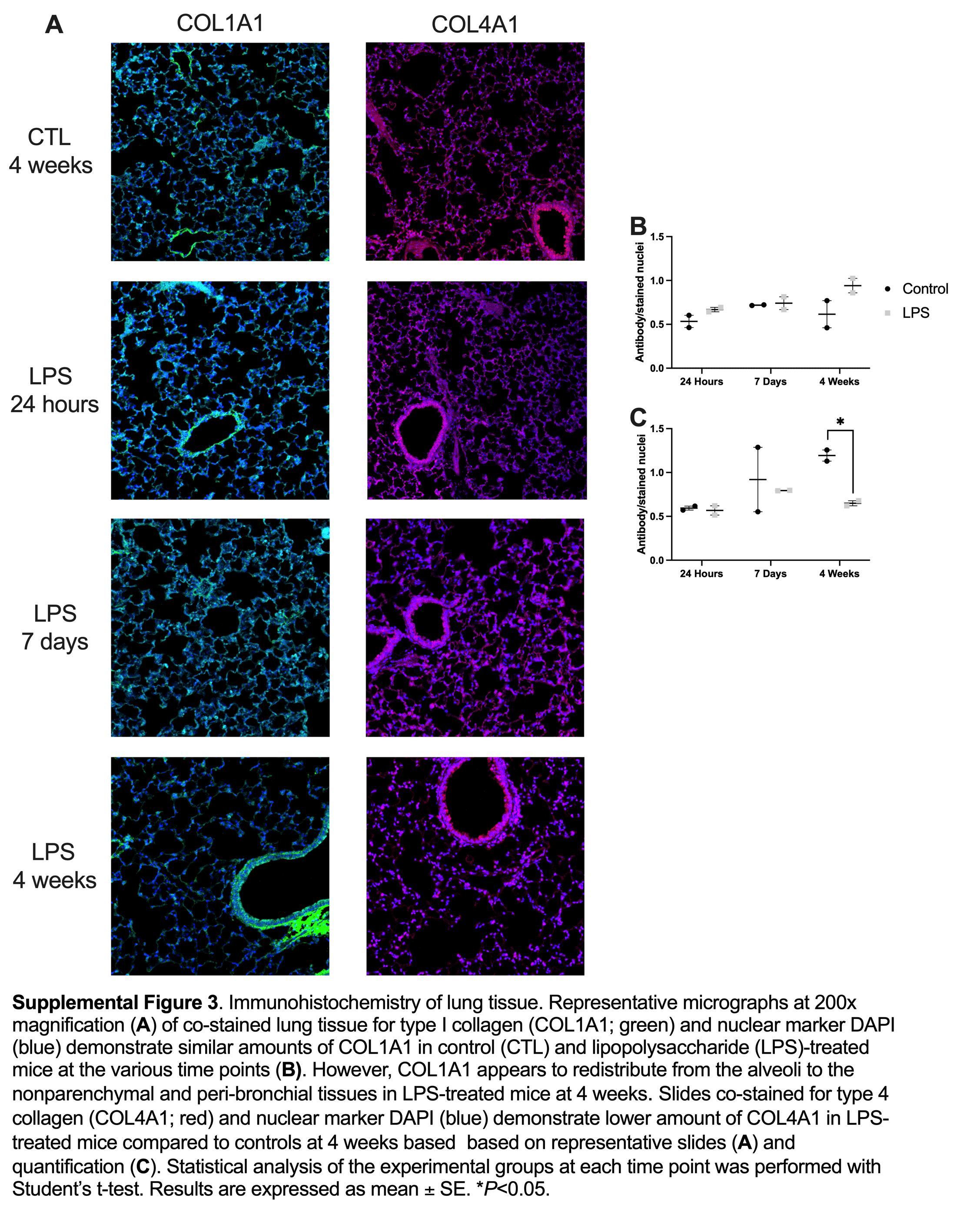

Supplement: Supplementary file 3 — Supplementary Information 3. [file 41598_2022_14618_MOESM3_ESM.tiff]
