# Supplementary material for: Lipopolysaccharide-induced murine lung injury results in long-term pulmonary changes and downregulation of angiogenic pathways
Source: Sci Rep. 2022 Jun 17;12:10245. doi: 10.1038/s41598-022-14618-8 (PMC9205148; doi:10.1038/s41598-022-14618-8)

Lipopolysaccharide-induced Murine Lung Injury Results in Long-term Pulmonary Changes and Downregulation of Angiogenic Pathways

Tsikis ST[1,2], Fligor SC[1,2], Hirsch TI[1,2], Pan A[1,2], Yu LJ[1,2], Kishikawa H[1,2], Joiner MM[1,2], Mitchell PD[3], Puder M*[1,2].

[1]Vascular Biology Program, Boston Children's Hospital, Harvard Medical School, Boston, MA 02115, USA
[2]Department of Surgery, Boston Children's Hospital, Harvard Medical School, 300 Longwood Ave, Fegan 3, Boston, MA 02115, USA
[3]Institutional Centers for Clinical and Translational Research, Boston Children's Hospital, Boston, MA 02115, USA

***Corresponding Author:**
Mark Puder, MD PhD
Boston Children's Hospital
Department of Surgery
300 Longwood Ave, Fegan 3
Boston, MA, 02115
Phone: 617-355-1838
Fax: 617-730-0477
Mark.Puder@childrens.harvard.edu

Supplementary Figure Legends and Files

Supplementary Figure Legends

Supplemental Figure 1. Percent change in weight from baseline at the various time points. Lipopolysaccharide (LPS)-treated mice had significantly greater weight loss compared to control mice at 24 hours (**A**), 4 days (**B**), and 7 days (**C**) after instillation. Both groups of mice had similar changes in weight at the 4 week (**D**) time point. Statistical analysis of the experimental groups at each time-point was performed with Student's t-test. Results are expressed as mean ± SE. ****P*<0.001; *****P*<0.0001.

Supplemental Figure 2. Bronchoalveolar fluid (BALF) differential cell counts. Lipopolysaccharide (LPS)-treated mice had significantly increased amounts of neutrophils compared to controls at 4 days after instillation **(A)**. Both groups had a similar number of neutrophils, monocytes, and lymphocytes at the 4 week time point **(B)**. Neutrophils were the predominant cell type in LPS-treated mice at 4 days while monocytes became predominant at 4 weeks (**C, D**). Monocytes were the predominant cell type in control mice at both time points (**C, D**). Total and differential cell counts of BALF were determined using the May-Grunwald-Giemsta stain (300 cells per animal). Each count was performed in biological duplicate. Statistical analysis was done using a two-way analysis of variance (ANOVA) model with interaction for group and cell type with Sidak adjustment for multiple comparisons (**C, D**). Results are expressed as mean ± SE. **P*<0.05, ***P*<0.01, ****P*<0.001,*****P*<0.0001.

Supplemental Figure 3. Immunohistochemistry of lung tissue. Representative micrographs at 200x magnification (**A**) of co-stained lung tissue for type I collagen (COL1A1; green) and nuclear marker DAPI (blue) demonstrate similar amounts of COL1A1 in control (CTL) and lipopolysaccharide (LPS)-treated mice at the various time points (**B)**. However, COL1A1 appears to redistribute from the alveoli to the nonparenchymal tissue in LPS-treated mice at 4 weeks. Slides co-stained for type 4 collagen (COL4A1; red) and nuclear marker DAPI (blue) demonstrate lower amount of COL4A1 in LPS-treated mice compared to controls at 4 weeks based on representative slides (**A**) and quantification (**C**). Statistical analysis of the experimental groups at each time point was performed with Student's t-test. Results are expressed as mean ± SE. **P*<0.05.

Supplemental File 1. Full length blots correspond to part of Figures 5A, 5C, 5E:

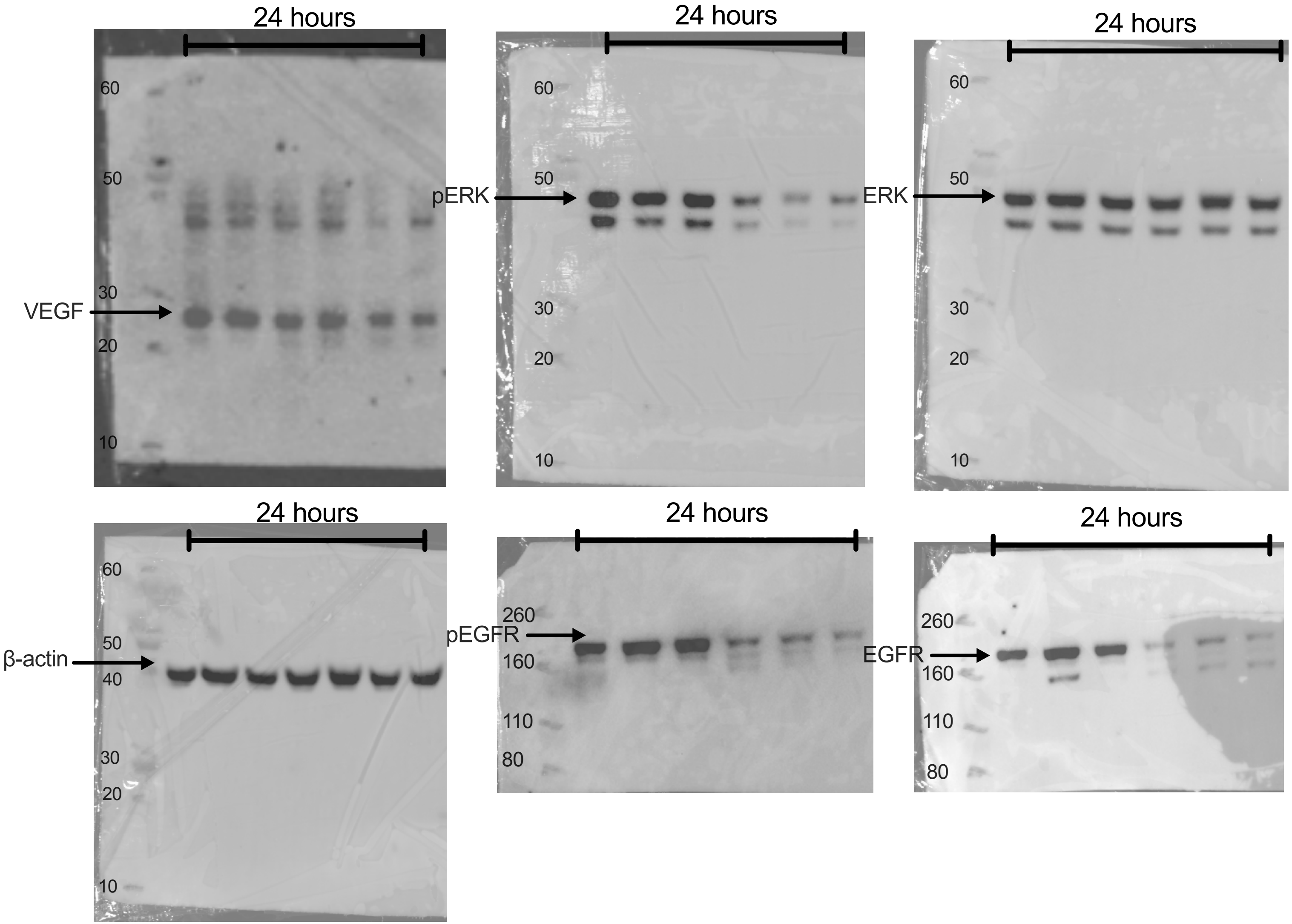

Supplemental File 2A. Full length blots correspond to part of Figure 5A:

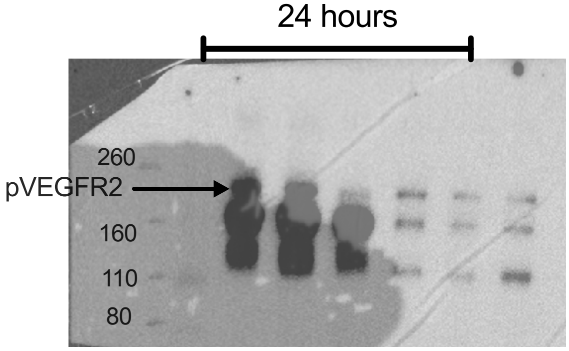

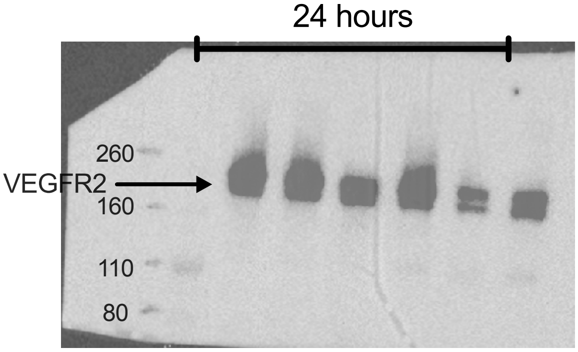

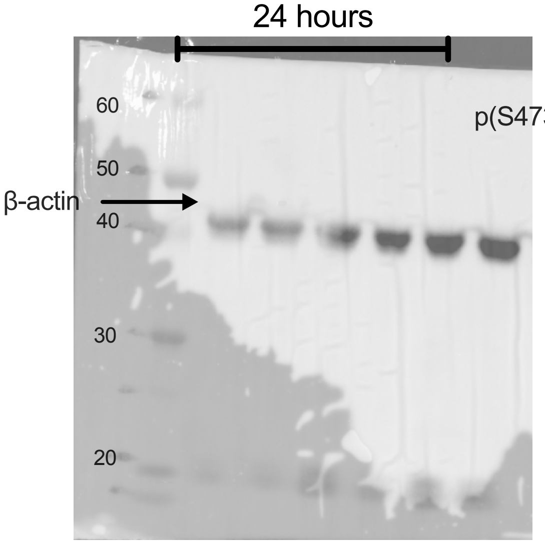

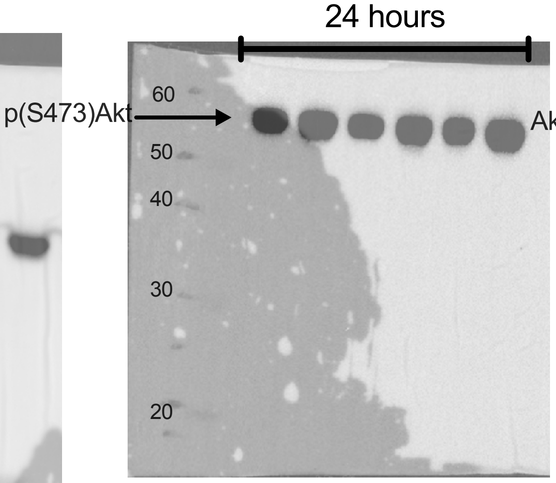

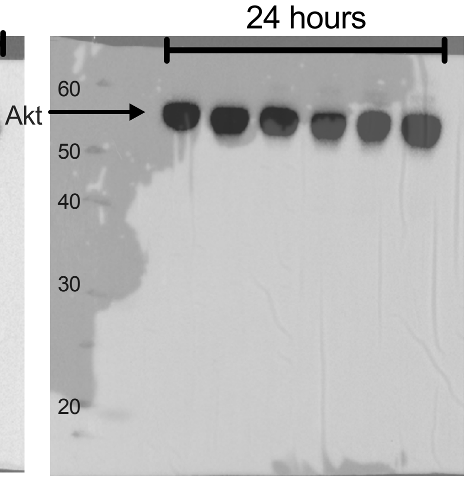

Supplemental File 2B. Full length blots correspond to part of Figure 5B:

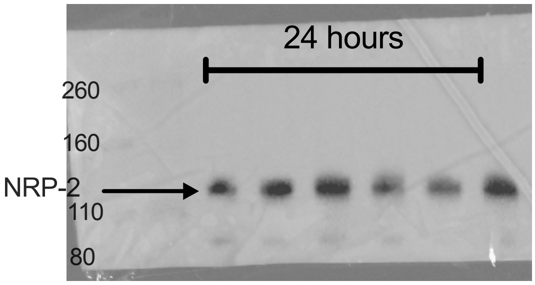

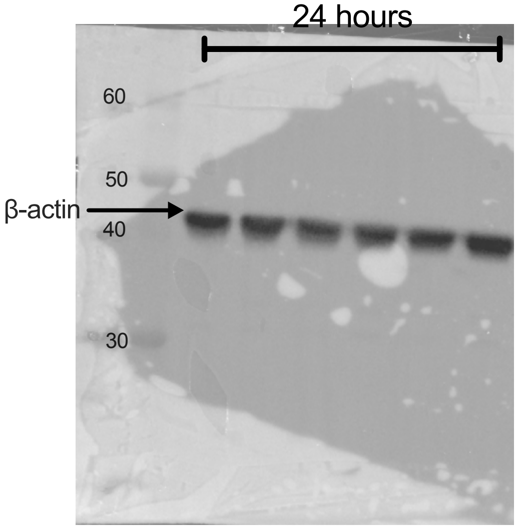

Supplemental File 3. Full length blots correspond to parts of Figure 5A, 5B, 5C, 5E:

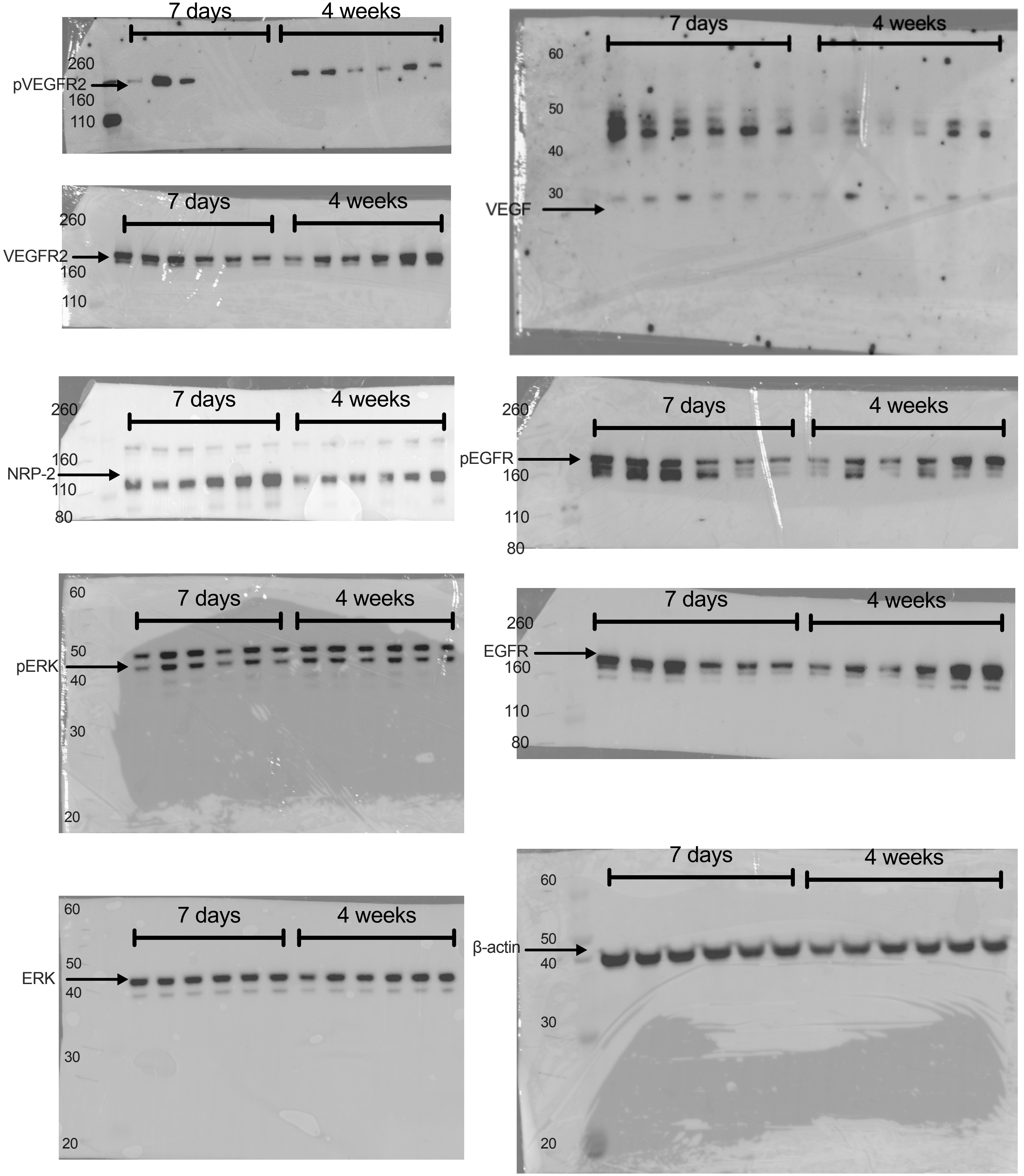

Supplemental File 4. Full length blots correspond to parts of Figure 5D:

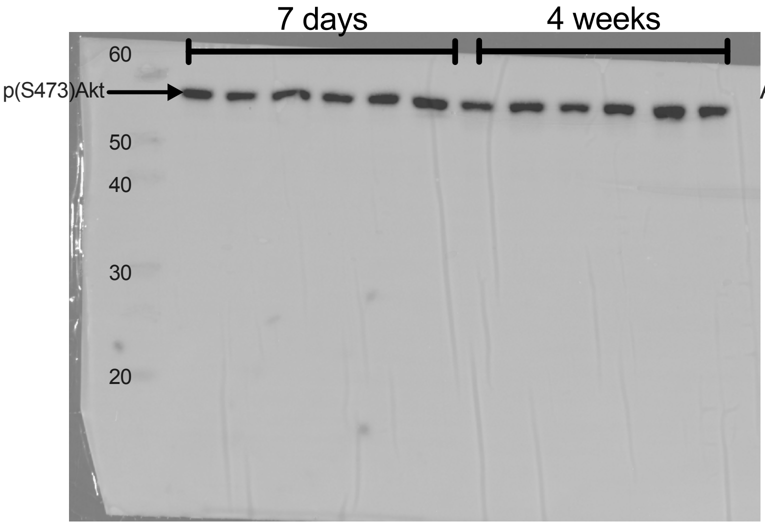

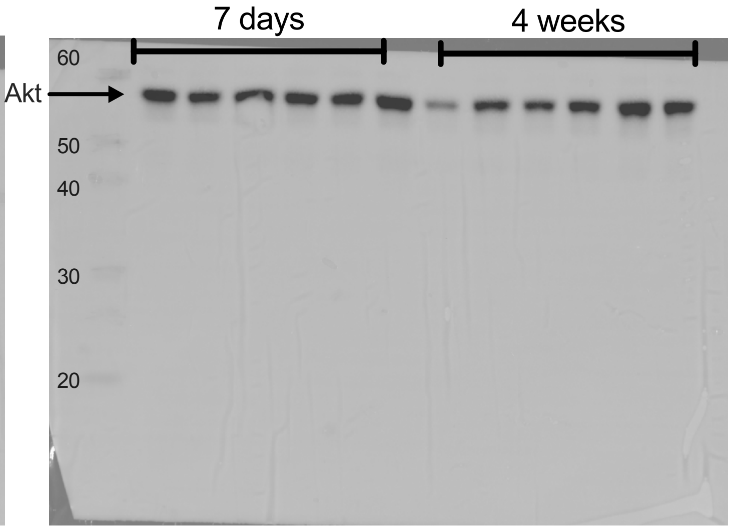

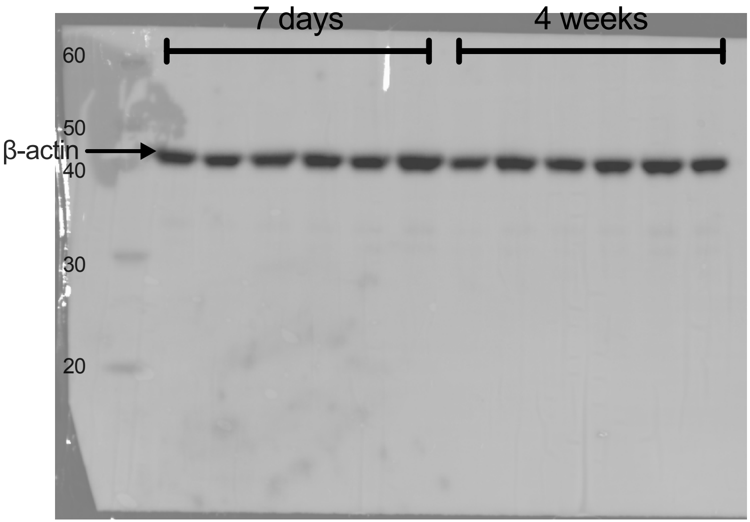

Supplement: Supplementary file 4 — Supplementary Information 4. [file 41598_2022_14618_MOESM4_ESM.pdf]
